# High-Risk HPV with Multiple Infections Promotes CYP2E1, Lipoperoxidation and Pro-Inflammatory Cytokines in Semen of Asymptomatic Infertile Men

**DOI:** 10.3390/antiox11061051

**Published:** 2022-05-26

**Authors:** Elvia Pérez-Soto, María Olivia Medel-Flores, Eduardo Fernández-Martínez, Rigoberto Oros-Pantoja, José Cruz Miranda-Covarrubias, Virginia Sánchez-Monroy

**Affiliations:** 1 Escuela Nacional de Medicina y Homeopatía, Instituto Politécnico Nacional, Mexico City 07320, Mexico; elvperezs@ipn.mx (E.P.-S.); molivof@ipn.mx (M.O.M.-F.); 2 Centro de Investigación en Biología de la Reproducción, Área Académica de Medicina del Instituto de Ciencias de la Salud, Universidad Autónoma del Estado de Hidalgo, Pachuca 42090, Mexico; efernan@uaeh.edu.mx; 3 Facultad de Medicina, Universidad Autónoma del Estado de México, Toluca de Lerdo 50180, Mexico; rorosp@uaemex.mx; 4 Hospital Militar de Especialidades de la Mujer y Neonatología de la Secretaría de la Defensa Nacional, Mexico City 11200, Mexico; cem@webmail.sedena.gob.mx; 5 Escuela Superior de Medicina, Instituto Politécnico Nacional, Salvador Díaz Mirón esq. Plan de San Luis S/N, Miguel Hidalgo, Casco de Santo Tomas, Mexico City 11340, Mexico

**Keywords:** human papillomavirus, CYP-2E1, pro-inflammatory cytokines, IL-4, lipid peroxidation, antioxidant enzymes, sperm morphology, male infertility

## Abstract

High-risk human papillomavirus (HR-HPV) infection, followed by chronic inflammation and oxidative stress, is a major risk factor of male infertility. In this study, we explored the potential impact of high-risk (HR) HPV genotypes in single infection (SI) and multiple infections (MI) that promote CYP2E1 expression, oxidative damage and pro-inflammatory cytokines, possibly contributing to sperm damage and male infertility. Semen samples from 101 infertile military men were studied. We analyzed seminal parameters, namely, HPV genotyping, cytochrome P450 2E1 (CYP2E1), oxidative stress biomarkers (total antioxidant capacity (TAC), catalase (CAT) and superoxide dismutase (SOD)), lipid peroxidation (LPO), 8-hydroxiguanosine (8-OHdG) and pro-inflammatory cytokines (IFN-γ, IL-1β, IL-4, IL-6 and IL-8). Eighty-one men (80.2%, 81/101) were positive for HPV infection, and MI-HR-HPV was higher than SI-HR-HPV (63% vs. 37%). HPV-52 was the most frequently detected type (18.5%), followed by HPV-33 (11.1%), and the most frequent combination of genotypes detected was HPV-33,52 (11.1%), followed by HPV-18,31 (6.2%). The group with infected samples presented lower normal morphology and antioxidant levels compared to non-infected samples. In concordance, the infected group showed high levels of LPO, IFN-γ, IL-1β, IL-4 and IL-6 and downregulation of CAT and SOD enzymes. Interestingly, changes in motility B, low levels of TAC, overexpression of CYP2E1, LPO and IL-8 levels were higher in MI-HR-HPV than SI-HR-HPV, suggesting that HPV infection promotes a chronic inflammatory process and a toxic and oxidative microenvironment, which increases with MI-HPV infections.

## 1. Introduction

Male infertility is considered multifactorial with various causes, including sexually transmitted infections (STI) such as human papillomavirus (HPV) [1,2,3,4] and *Chlamydia trachomatis* [5,6], as well as chronic inflammation [7] and oxidative stress (OS) [7,8]. However, the mechanisms associated with these changes in infertile men have not yet been fully described.

Concerning HPV infection, our group, Pérez-Soto et al., 2021, recently demonstrated that HPV infection can increase lipoperoxidation (LPO), 8-hydroxydeoxyguanosine (8-OHdG) and even pro-inflammatory cytokines such as IFN-γ, IL-1β and IL-6 in semen of infertile men [5].

Overexpression of the cytochrome P450 2E1 (CYP2E1) enzyme has been detected in HPV-immortalized oral and cervical epithelial cultures [9], suggesting that changes in the microenvironment by circulating toxic metabolites may be modulated by HPV. Moreover, spermatogenesis disorders and the overexpression of CYP2E1 mRNA have been correlated in male rats with experimental alcoholism, type I diabetes, and obesity [10,11]. Therefore, in this study, we focused on CYP2E1, which is the most important enzyme in xenobiotic metabolism [12]. Many CYP2E1-catalyzed reactions not only generate reactive metabolites but also produce reactive oxygen species (ROS), such as superoxide and hydrogen peroxide [13], which can damage sperm, because the high content of polyunsaturated fatty acids (PUFAs) in the sperm plasma membrane makes them susceptible to lipid peroxidation in the presence of oxidative stress (OS) [14]. Moreover, it has been reported that spermatogenesis and fertility are influenced by PUFA content in the sperm plasma membrane [15]. We therefore set out to evaluate the potential impact of HR-HPV genotypes in single infection (SI) and multiple infections (MI) that promote CYP2E1 expression, oxidative damage and pro-inflammatory cytokines, which can then contribute to sperm damage and male infertility.

## 2. Materials and Methods

### 2.1. Study Design and Population

A cross-sectional study was designed in which semen was collected from 101 male military members; this population was selected during infertility consultations between January 2016 and November 2019 at the “Hospital Militar de Especialidades de la Mujer y Neonatología” of the Secretaría de la Defensa Nacional (SEDENA), Mexico City.

Inclusion criteria were men attending the hospital for conjugal infertility investigation who failed to conceive after 12 months or more of regular unprotected sexual intercourse and who agreed to be subjected to seminology laboratory tests at the hospital. Standard function tests were carried out on each sample, including details of sperm number, motility and morphology. The criterion of cellularity in semen was defined as containing more than 1 × 10^6^/mL of white blood cells, as detailed by the World Health Organization (WHO). Additionally, a clinical examination was conducted. Men undergoing drug therapy and those with undescended testes, varicocele or other structural abnormalities were excluded.

Ethical approval was granted by the Institutional Human Research Ethical Committee of the hospital (19-CI-09-016-025). All the patients gave their informed consent according to the Helsinki Declaration and the Official Mexican Standard (NOM-012-SSA3-2012).

Semen samples were obtained by masturbation after two to seven days of sexual abstinence and allowed to liquefy for 30 min at 37 °C. The sperm parameters were evaluated according to the current WHO [16].

Semen samples were centrifuged at 300× *g* for 10 min, and seminal plasma and cell pellets were separated and stored at −20 °C until analysis.

### 2.2. Virus Detection and Genotyping

DNA extraction from semen samples was performed using the DNeasy Blood and Tissue Kit (Qiagen, UK), and then viral genome detection was performed by polymerase chain reaction (PCR) using universal primers MY09/11, GP5+/6+ and L1C1/C2 [17]. The β-globin gene was used as a positive control for DNA extraction and PCR methods. All HPV-positive samples were also analyzed for the detection of genotypes using the MPCR Kit for Human Papilloma Virus Set 2 (Maxim Biotech Inc., San Francisco, CA, USA). The kit is based on multiplex PCR, which simultaneously amplifies HPV genotypes classified into two major groups, low-risk (LR) types such as HPV-6 and -11, and high-risk (HR) types or carcinogens such as HPV-16, -18, -31, -33, -52 and -58, depending on the ability of the virus to induce malignant transformation [18].

### 2.3. Oxidative Stress (OS) Biomarkers in the Seminal Plasma

#### 2.3.1. Relative Gene Expression of CYP2E1 Enzyme and Antioxidant Enzymes Catalase (CAT) and Superoxide Dismutase (SOD) by qRT-PCR

The relative gene expression of CYP2E1, CAT and SOD was evaluated using real-time quantitative reverse transcription PCR (qRT-PCR).

Total RNA was isolated from semen samples using Trizol according to the manufacturer’s protocol (Ambion, Life Technologies, Carlsbad, CA, USA). Then, RNAs were treated with DNase I (Promega, Madison, WI, USA), and cDNA synthesis was performed using the SuperScript First-Strand Kit (Invitrogen, Life Technologies, CBAD, CA, USA). The GAPDH was used as an endogenous gene. qRT-PCR was performed in Stratagene Mx3005 (Agilent Technologies, Santa Clara Valley, CA, USA) using SYBR Green PCR Master Mix (Applied Biosystems, Waltham, MA, USA). Specific primers were designed by using Primer Express version 3.0.1 software qRT-PCR: CYP2E1 forward: (5′ AGAGATGCCCTACATGGATGCT 3′), CYP2E1reverse: (5′ GGGCACGAGGGTGATGAAC 3′) CAT forward: (5′TTACTCAGGTGCGGGCATTC 3′), CAT reverse: (5′ CGTAGTCAGGGTGGACCTCAGT 3′), SOD forward: (5′ ATGGTGTGGCCGATGTGTCT 3′), SOD reverse: (5′ TTCCAGCGTTTCCTGTCTTTG 3′) GAPDH forward: (5′ CATTCATTGACCCGGAATACATG 3′) GAPDH reverse (5′ GGATCTGTTTGGGCTCTTTGC 3′). Relative expression was calculated employing the 2^−∆∆CT^ method [19].

#### 2.3.2. Quantification of Total Antioxidant Capacity (TAC)

The non-enzymatic TAC of seminal plasma was determined by the ferric reducing of antioxidant power (FRAP) method described by Benzie [20], with minor modifications [5]. Finally, the absorbance values of blank, standards (100, 250, 500 and 1000 µM/L FeSO_4_) and samples were estimated by a spectrophotometer at 593 nm. The results were corrected for dilution and are expressed as µmol FeSO_4_/L. All solutions were freshly prepared and immediately used. Measurements were performed in triplicate.

#### 2.3.3. Assessment of Lipoperoxidation

LPO in seminal plasma was determined by the measurement of malondialdehyde (MDA) using the thiobarbituric acid (TBA) test, with minor modifications [5,21]. The supernatant was read on a spectrophotometer (Thermo Fisher Scientific, Waltham, MA, USA) at 532 nm. The results are expressed as nanomoles of MDA per milligram of protein. Protein was determined according to the method described by Bradford using bovine serum albumin as a standard.

#### 2.3.4. 8-Hydroxydeoxyguanosine Assay

DNA oxidation was evaluated by the formation of 8-hydroxydeoxyguanosine (8-OHdG), a ubiquitous marker of OS. The levels of 8-OHdG in 100 µL of seminal plasma were measured using the OxiSelect Oxidative DNA Damage ELISA Kit (Cell Biolabs Inc., San Diego, CA, USA). The absorbance of each sample was determined in a microplate reader (Thermo Fisher Scientific, Waltham, MA, USA) at a wavelength of 450 nm, and the levels of 8-OHdG were calculated from a standard curve. The minimum detectable concentration of 8-OHdG was 20 ng/mL and the maximum detectable concentration was 100 ng/mL.

### 2.4. Cytokine Expression by Enzyme-Linked Immunosorbent Assays (ELISA)

Cytokine concentrations were measured in seminal plasma (dilution 1:1), using specific commercial Enzyme-Linked Immunosorbent Assay (ELISA) kits (PeproTech, Rocky Hill, NJ, USA). The measurement ranges of the cytokines were as follows: IFN-γ, 8–3000 pg/mL; IL-1β, 8–1000 pg/mL; IL-4, 16–1000 pg/mL; IL-6, 24–1500 pg/mL; IL-8, 16–1000 pg/mL. The standard curves were always the same, indicating that seminal plasma does not interfere with cytokine detection in this assay system. Seminal cytokine concentrations are expressed as pg/mL of seminal plasma.

### 2.5. Statistical Analysis

The frequencies of HPV and genotypes were expressed as frequency (%). Descriptive statistics were used for the comparison of seminal parameters, OS biomarkers and cytokines in the semen. The data are expressed as means ± standard error (SD) for normal distribution and median ± [25th, 75th] percentiles for abnormal distribution. Kolmogorov–Smirnov’s test was used to verify normality and a Levene’s test was used to verify the homogeneity of variances. To compare two groups, the nonparametric statistical Mann–Whitney test was applied. To compare more than two groups, the parametric statistical test one-factor analysis of variance (ANOVA) with post hoc Tukey’s and the nonparametric statistical test Kruskal–Wallis one-way analysis of variance of ranks with post hoc (K–W test) were applied. Spearman’s coefficient R for nonparametric correlation was calculated in HPV-infected groups. In all cases, statistical significance was considered when *p* < 0.05. Data were analyzed using SPSS Statistical Software, version 24 (SPSS Inc., Chicago, IL, USA), and Microsoft Excel (Windows 10).

## 3. Results

### 3.1. Detected HPV Genotypes in the Population

A total of 101 men ranging in age from 29 to 69 years old participated in the study. The HPV infection rates in semen samples are summarized in Table 1. Overall, 80.2% (81/101) of the study population was HPV-positive. Single HPV infections occurred in 37% of HPV-positive semen samples. Concerning genotypes for SI, we detected that the oncogenic HR-HPV genotypes were higher than LR-HPV genotypes (33.3% vs. 3.7%), and the HPV52 genotype was the most frequent (18.5%), followed by HPV-33 (11.1%). On the other hand, infections with multiple genotypes of HPV occurred in 63% of HPV-positive semen samples. HPV in MI exclusively with high-risk HPV genotypes occurred in 35.8% (29/81), while the remaining samples were with HPV in MI with LR-HPV and HR-HPV genotypes detected simultaneously with 27.2% (22/81), and the most frequent combination of genotypes detected was HPV33,52 (11.1%), followed by HPV18,31 (6.2%).

Considering that 96.3% of genotyping was HR oncogenic HPV infection in semen samples, the results were organized in the groups SI-HPV or MI-HPV, all with high-risk HPV.

### 3.2. Seminal Parameters in the Study Groups

The results of the seminology laboratory tests showed that seminal parameters were normal in the study population, according to WHO-5 (World Health Organization, 2010), except for the percentage of normal morphology, with a median of 2.5% and an interquartile range of 3% in the study population. Table 2 summarizes sperm parameters for the study population from the non-infected HPV group and the two infected groups, SI-HPV and MI-HPV. There were significant differences regarding the percentage of normal morphology between the non-infected HPV group and the infected groups, and in low progressive motility (%B) between the non-infected group and the MI-HPV group.

### 3.3. Oxidative Damage in the Semen of Infertile Men

#### 3.3.1. Relative Gene Expression of Cyp2E1

**Figure 1:**
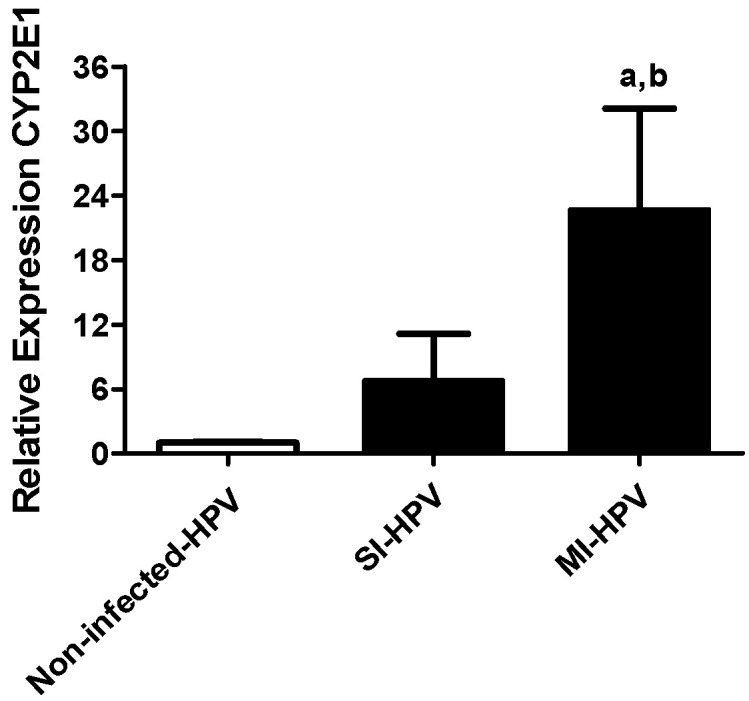
Relative gene expression to CYP2E1 in study groups. Relative expression of CYP2E1 in the non-infected group and groups with single infection (SI-HPV) or multiple infections (MI-HPV). Data are expressed as the means ± SD and were analyzed by one-way ANOVA. Significant difference was defined as *p* < 0.05. ^a^ Infected HPV group vs. non-infected group; ^b^ SI-HPV group vs. MI-HPV group.

Relative gene expression of CYP2E1 showed a seven- and 22-fold overexpression in the SI-HPV and MI-HPV groups, respectively, compared to the non-infected group (Figure 1). Interestingly, we also detected a three-fold overexpression in MI-HPV compared to SI-HPV.

#### 3.3.2. Lipoperoxidation and DNA Damage in the Semen

The LPO and 8OH-dG are summarized in Table 3. The level of LPO was significantly higher in males with HPV infection, and also in the MI-HR-HPV group compared to the SI-HR-HPV group (*p* < 0.05). Unexpectedly, the 8OH-dG level did not show any differences between groups, although the DNA damage level was high in all groups in comparison with values of around 1.2 ng/mL described in healthy fertile men [22].

#### 3.3.3. Antioxidants in the Semen

**Figure 2:**
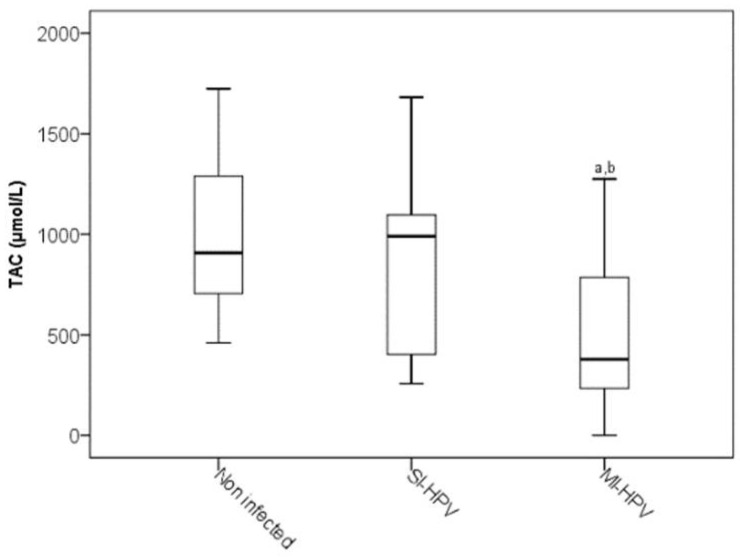
Total antioxidant capacity in study groups. The significance test is the Kruskal–Wallis test for non-distributed variables (*p* < 0.05). ^a^ Infected HPV group vs. non-infected group; ^b^ SI-HPV group vs. MI-HPV group.

Concerning the non-enzymatic marker of TAC, MI-HPV infection showed a significant decrease with respect to the non-infected HPV group and SI-HPV group (*p* < 0.05), (Figure 2).

**Figure 3:**
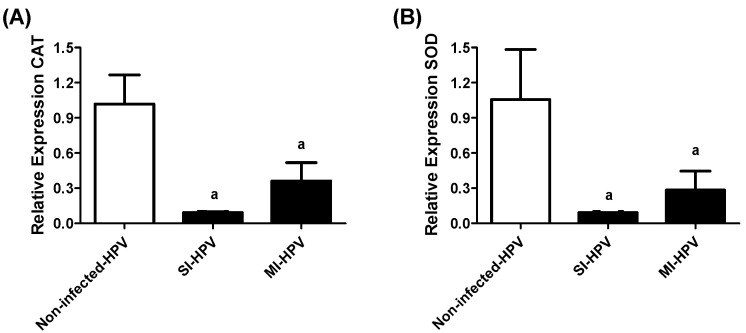
Relative gene expression of CAT and SOD in study groups. (**A**) Relative gene expression of catalase (CAT), (**B**) superoxide dismutase (SOD) in non-infected group, and groups with single infection (SI-HPV) or multiple infections (MI-HPV). Data are expressed as the means ± SD and were analyzed by one-way ANOVA. Significant difference is defined as *p <* 0.05. ^a^ Infected HPV group vs. non-infected group.

The enzymatic antioxidant systems of CAT and SOD mRNA were evaluated by qRT-PCR. The results showed a downregulation of both enzymes in infected groups compared to the non-infected group (*p* < 0.05). We detected an 11-fold downregulation of CAT and SOD in SI-HPV in comparison with the non-infected group. We similarly detected downregulations of 3- and 4-fold of CAT and SOD in MI-HPV compared to the non-infected group, respectively, as shown in Figure 3. No differences were detected in the expression of both enzymes between SI-HPV and MI-HPV. Thus, HPV infection alters the expression of antioxidant enzymes in semen.

### 3.4. Correlation between OS Biomarkers and Sperm Morphology in the Infected Population

In this study, evaluations of the levels of OS biomarkers relating to the sperm morphology and defects in spermatozoa were performed for the infected population (Table 4). Spearman’s correlation showed a positive correlation between CYP2E1 and SI-HPV and MI-HPV groups and LPO (*R* = 0.841 and 0.323, *p* < 0.05), and defects in the tail and 8OH-dG (*R* = 0.246, *p* < 0.05). By contrast, the levels of CYP2E1 in seminal plasma negatively correlated with TAC (*R* = −0.411, *p* < 0.05), 8OH-dG negatively correlated with % normal morphology, and TAC negatively correlated with defects in the intermediate piece (*R =* −0.283, *p* < 0.05).

### 3.5. Seminal Pro-Inflammatory Cytokines

Table 5 shows the seminal cytokines determined in the study groups. The levels of pro-inflammatory cytokines (IFN-γ, IL-1β, IL-4, IL-6) in seminal plasma were significantly higher in infected groups than the non-infected group (*p* ≤ 0.05). Finally, the concentration of IL-8 was different in the non-infected HPV group compared to the MI-HR-HPV group (*p* ≤ 0.05).

## 4. Discussion

To the best of our knowledge, this is the first study to detect and demonstrate single and multiple infections with HR-HPV genotypes and their effects on the oxidative and inflammatory environment and sperm quality in infertile men. We detected an overall HPV prevalence of 80.2% (81/101), which is the highest found in studies of infertile men. A recent meta-analysis in infertile populations described a 16% level of HPV from semen samples [2], similar to a study in Brazil with 16.6% (38/229) [23] in infertile couples, up to a level of 35.7% in unexplained infertility [1]. The results of this study reveal that HPV infection in semen is rather common, showing a relatively high prevalence in asymptomatic young male partners of infertile couples of the military community in Mexico.

Our study found that the most common type overall is HPV-52, which accounted for 18.5% (15/81) of semen samples in Mexican asymptomatic infertile men of the military community, similar to Jeršovienė et al., who also found that HPV-52 is the most frequent type in 25% of all semen samples in male partners of infertile couples in Lithuania [4], and Yang et al., who found an HPV-52 prevalence of 8.19% in Chinese infertile men [24]. In addition, the prevalent genotypes in SI-HPV and MI-HPV were, in decreasing order, HPV-52 (42/81), HPV-33 (26/81) and a combination of HPV-33,52 (15/81) in all semen samples in this study, and we found a higher prevalence of HR-HPV infection compared to another study of infertile Mexican men, which reported 27.27% (6/22) of HPV infection, and in which, notably, HR-HPV genotypes (HPV-18, -45, -51, -52 and -54) were also detected [25]. Therefore, HPV-52 is the most prevalent genotype in Mexican asymptomatic infertile men of the military community.

Regarding HPV groups, the prevalence of SI with exclusively HR genotypes was similar to Damke et al., 2017 (33.3%, 27/81 vs. 34.2%, 13/38). Furthermore, in this work, we found MI with exclusively HR-HPV genotypes (35.8%, 29/81), similar to Gimenes et al., who found MI of HR-HPV types in 41.1% (7/17) [6]. On the other hand, HPV multiple infection was higher than reported by Damke et al. (63.0%, 51/81 vs. 36.8%, 14/38) [23], while samples with MI-HPV with low-risk and high-risk HPV types detected simultaneously showed fewer cases compared with Damke et al. (43.1%, 22/51 vs. 64.3%, 9/14) [23], with HR-HPV genotypes predominating in our results (56.9%, 29/51).

The results reported here are so far the highest documented in a population of asymptomatic infertile military men. The high prevalence in our study could be due to the specific population of asymptomatic infertile military patients, who may be affected by other viral or bacterial infections, as described in a few previous studies [5,6,25]. This risk factor of infections with other pathogens not detected may also contribute to dysregulation in male reproductive function and fertility, owing to obstruction or sub-obstruction, altered secretory function and the release of inflammatory mediators [26]. There are other risk factors such as risky sexual behavior, a large number of sexual partners and anal intercourse, which increase the risk of acquiring and transmitting an STI, including HPV infection in males [27]. Additionally, military status contributes to the development of psychological, endocrinological and immunological alterations due to stress [28,29]. Such conditions can increase the susceptibility to chronic HPV infections.

The effect of SI or MI with HPV types on sperm quality and male infertility is controversial, although in most reports, there is an alteration of sperm parameters such as motility, sperm concentration, pH, leukocytes and sperm morphology [2,4,22,23,30]. The results of the current study indicate that the normal morphology in groups infected was lower than in the non-infected HPV group (*p* < 0.05, K–W test), as reported by other authors [24,30]. Recently, Tavakolian et al. (2021) found the same relationship: in nine patients positive for HPV infection, the percentage of morphology was significantly decreased compared to the negative control group with normospermia (6.3 ± 0.7% vs. 2.3 ± 0.6%; *p* = 0.023) [31]. Relevant to this question, in this study, the occurrence of SI and MI with HR-HPV genotypes decreases the normal morphology percentage, and it is correlated with DNA damage, favoring sperm defects (% defects of head followed by tail and intermediate piece). Yukki et al. (2021) analyzed that in 6.9% (15/216) of samples positive for HR-HPV genotypes (HPV-16, -18, -33, -35, -39, -45, -51, -52, -58, -68), the HR-HPV infection was located in the head and intermediate piece of sperm of infertile Japanese men [32]. On the other hand, progressive motility (A+B) was not diminished with respect to the WHO criteria [16], but in the MI-HPV group, the low progressive motility (%B) was significantly increased, so the motility begins to be impaired by defects in the intermediate piece and mitochondria by excessive free radicals in the seminal microenvironment. In other studies, HPV infection causes decreased motility [23,25,33]. In this study, we detected increased morphological defects mainly in the tail, correlated with OS. As the role of the sperm tail is to produce motility, alterations decrease sperm motility [33]. However, sperm motility was not reduced in conditions of OE, as has been described in other reports [34]. One possible explanation is that the subjects explored here were young and probably have better resistance to oxidation, as it has been demonstrated that the percentage of motile spermatozoa decreases with age [35]. Moreover, lifestyle practices linked with antioxidant protection in this population and the existence of additional non-studied mechanisms that connect the activity of OE and motility, such as peroxiredoxins, glucose 6-phosphate dehydrogenase and paraoxonase-1 activity, which also have important roles in antioxidant protection [34,35], could explain this result.

In relation to CYP2E1 expression, the results demonstrate that the highest signal found in this study by HPV infection was the overexpression of CYP2E1, and moreover, a differentially higher expression was detected in MI-HPV compared to SI-HPV. Concerning this finding, a recent study showed that men with seminal HPV DNA had higher relative abundance of specific seminal bacteria compared to men without seminal HPV DNA [36]. In this study, the overexpression of CYP2E1 could be caused by metabolites generated by co-infection with other microbes, as has been described in previous studies [5,6,25], and the higher overexpression in MI over SI could be explained by the higher abundance of HPV diversity and a higher abundance of resident microbes. In studies of microbial metabolites and HR-HPV in women, it has been demonstrated that H_2_O_2_ and HR-HPV were significatively correlated [37] along with higher biogenic amines and lower glutathione and lipid concentrations [38]. In addition, it has recently been shown that in human sperm, alterations in water and hydrogen peroxide diffusion are related to a direct interaction of viral L1 protein of HPV with aquaporin-8 [39].

On the other hand, a previous report of Farin et al., 1995, demonstrated the capacity of specific HPV-16 to act in concert with cellular changes by the overexpression of CYP2E1 in oral and cervical epithelial cells in vitro [9], similarly to the CYP2E1 overexpression detected here; this may be due to the contribution of type HPV-16, which was detected in both study groups, although it predominated in the MI-HPV group (18.3% in MI-HPV vs. 1.2% in SI-HPV).

Moreover, spermatogenesis abnormalities and overexpression of CYP2E1 mRNA in male rats have also been correlated in experimental alcoholism, type I diabetes and obesity models [10,11], suggesting that a possible mechanism of these pathologies is the contribution to a high level of free radical production (ROS and RNS) and, consequently, an effect on spermatogenesis due to LPO and chromatin integrity. Similarly, in this study, CYP2E1 correlates positively with LPO and negatively with TAC levels in the presence of HPV infection (*p* < 0.05).

In relation to the comparison of antioxidant signals between groups, we found that infected samples had lower CAT and SOD expression and TAC activity levels than non-infected samples. This finding demonstrates a higher OS state in the HPV-infected groups, and the ineffective defense of antioxidants against superoxide, H_2_O_2_ and other free radicals.

In addition, the TAC level was lower in MI-HPV compared to SI-HPV, suggesting a higher OS level in MI-HPV than SI-HPV, in concordance with CYP2E1 results. Our previous report also demonstrated a low TAC level in semen samples with HPV infection [5]. Moreover, in this study, the influence of MI-HPV was detected, indicating a higher OS in MI-HPV than SI-HPV and more depressed TAC activity, which is dependent on enzymatic antioxidants, and nonenzymatic antioxidants by the overproduction of ROS, and RNS thus disrupts cellular functions and leads to male infertility.

In concordance with low antioxidant signals, evidence of oxidation of lipids and nucleic acids was also detected. We detected a higher level of LPO in infected groups, and interestingly, its concentration was twofold higher in the MI-HPV group over the SI-HPV group, indicating major damage due to multiple infections. LPO is a result of a high level of OS. A high LPO changes the accumulation, structure and dynamics of lipid membranes, and since these lipids are highly reactive compounds, they can produce ROS [40].

OS leads to the production of oxidized DNA base compounds, such as guanine oxidized to 8-OHdG. Several studies have shown that 8-OHdG is the best biomarker of DNA oxidation in the sperm of infertile men [22,41]. In this study, we detected high values of 8-OHdG, and the mean values were higher in this military population (7.94 ± 1.27 ng/mL) compared with other infertile male populations (3.2 ± 0.13 ng/mL) [22]. However, no significant differences between non-infected and infected groups were detected, suggesting that different factors of infection, such as genetic, physical, psychological and lifestyle-related factors may have contributed to DNA damage.

Moreover, inflammation and OS are closely linked pathologies and establish a vicious loop impairing male fertility [7,8,42], including HPV [5]. The cytokines and chemokines play important roles in fertility and infertility [43]. Furthermore, they participate in the reproduction, innate and cellular immune responses, inflammation, growth regulation, germ cell differentiation and intracellular transduction signals in the male reproductive tract [44,45,46]. Cytokines are contained in the seminal plasma, since they are part of the autocrine/paracrine network operating in the male tract to activate the innate host defense to combat genital tract pathogens by secretion of pro-inflammatory cytokines such as interleukins (IL-1β, IL-6), tumor necrosis factor (TNF-α), interferons (IFN-γ), chemokines (IL-8) and anti-inflammatory cytokines (IL-4, IL-10) [47,48,49] by immunocompetent cells distributed in different parts of the genital tract and other components [47]. The imbalance of some cytokines such as IL-4 can cause male infertility [50].

In this work, similar to our previous report, we demonstrated that HPV infection promotes chronic inflammation through high levels of IFN-γ, IL-1β and IL-6 [5] caused by single or multiple infection, and even an increment of IL-4, as in a preliminary study [50]. Furthermore, multiple infections, all with HR-HPV genotypes, even induce IL-8 and other metabolic cytotoxic by-products that trigger higher LPO than the SI-HPV group, so there is more cytotoxic damage, which results in a high expression of CYP2E1, affecting the total and enzymatic antioxidant capacity. This effect is caused by the overproduction of free radicals (ROS and RNS) by inflammatory cells (which are among the major sources of ROS/RNS) at the location of the inflammation, thus disrupting cellular functions and leading to a variety of pathologies, including male infertility [7].

Moreover, Th2 cells, augmented by the HR-HPV genotypes, alter the IL-4 and JAK-STAT signaling, and this leads to male reproductive dysfunction, and even to ROS or RNS activating an intracellular signaling cascade that elevates the pro-inflammatory gene expressions and other molecules such as CYP2E1. Thus, OS and inflammatory pathways operate in loops, each triggering the other by HPV infection.

On the other hand, Georgescu et al. also documented that the pathogenesis of HPV infection is associated with chronic inflammation and oxidative stress, all cofactors involved in carcinogenesis [18]. Moreover, HPV often occurs in the semen of patients with male accessory gland infection (MAGI) [51], and patients with persistent HR-HPV genotype have signs of prostatitis, which is associated with prostate cancer (PCa) [52]. In addition, the increased risk of malignancy [53] and metabolic diseases has been associated with elevated CYP2E1 expression [10,11], as was found in this study. It is therefore important to attend to infection to prevent the development of PCa, which has been associated with HPV infection [54].

This work had limitations in design and included only a small sample size, so we visualize that a case and control study is required (fertile and infertile men) with a greater number of participants. Studies in progress including healthy fertile controls in the general population and military communities could confirm our findings. We need to repeat HPV screening in the semen after 12 months to see if there is clearance or persistence of seminal HPV infection. However, the effect of seminal HPV infection on male oxidative stress infertility cannot be ignored. Therefore, the findings should be carefully interpreted to diagnose and treat infection and OS in male partners of infertile couples.

## 5. Conclusions

The results presented here show that HPV infection promotes a chronic inflammatory process and a toxic and oxidative microenvironment which increases with MI-HPV infection.

Therefore, the detection of HR-HPV infection genotypes, pro-inflammatory cytokines and OS biomarkers may be useful complementary tools for male infertility diagnosis, even to propose new perspectives on its pathogenesis (NF-kB and NRF-2), therapeutic approaches (vaccines, antiviral, immunomodulators and antioxidants) and the prevention of the development of other diseases.

## Figures and Tables

**Table 1:** Detected HPV genotypes in semen of the HPV-positive samples in the population.

Group	HPV Genotypes	*n*	Percentage (%)
Single infection (SI-HPV)	HPV52	15	18.5
	HPV33	9	11.1
	HPV31	2	2.5
	HPV6	2	2.5
	HPV16	1	1.2
	HPV11	1	1.2
		30	37.00%
Multiple infection (MI-HPV, all with HR-HPV)	HPV 33, 52	9	11.1
	HPV 18, 31	5	6.2
	HPV 16, 31, 33, 52	3	3.7
	HPV 31, 52	2	2.5
	HPV 16, 18	1	1.2
	HPV 16, 31	1	1.2
	HPV 18, 33	1	1.2
	HPV 18, 52	1	1.2
	HPV 18, 58	1	1.2
	HPV 33, 51	1	1.2
	HPV 16, 18, 33	1	1.2
	HPV 16, 33, 52	1	1.2
	HPV 16, 31, 52	1	1.2
	HPV 33, 52, 58	1	1.2
		29	35.80%
Multiple infection (MI-HPV, all with low and high HPV)	HPV 18, 6	6	7.4
	HPV 6, 52	3	3.7
	HPV 11, 16	2	2.5
	HPV 6, 18, 52	2	2.5
	HPV 6, 11, 52	2	2.5
	HPV 6, 11, 16	2	2.5
	HPV 11, 16, 58	1	1.2
	HPV 11, 16, 52	1	1.2
	HPV 11, 33, 52	1	1.2
	HPV 6, 11, 16, 18, 58	1	1.2
			27.20%

Percentage not summed to total due to rounding; HPV: human papillomavirus; DNA: deoxyribonucleic acid.

**Table 2:** Association between HPV positivity and semen parameters and CT positivity (*n* = 101).

Test	Non-Infected HPV ^1^ (*n* = 20)	SI-HPV ^2^ (*n* = 30)	MI-HPV ^3^ (*n* = 56)	*p*
Volume (mL)	2.65 ± 1.46	3.37 ± 1.59	3.11 ± 1.21	0.142
pH	8 [7.50, 8]	8 [7, 8]	8 [7, 8]	0.135
Total sperm number (×10^6^/ejaculate)	56 [38.0, 133.5]	93 [38.7, 95]	94 [51, 162.5]	0.332
Sperm concentration (×10^6^/mL)	28.75 [14.65, 66.00]	28.5 [8.25, 76]	27.5 [18, 57]	0.819
Normal morphology (%)	5 [3, 7]	2 [1, 3] ^a^	2 [1, 4] ^a^	0.004
Abnormal sperm morphology (%)	95 [97, 99]	98 [97, 99] ^a^	98 [97, 99] ^a^	0.004
Head defects (%)	43.93 ± 11.20	42.82 ± 11.03	41.71 ± 12.30	0.737
Midpiece defects (%)	22.80 ± 13.04	24.82 ± 9.09	24.31 ± 7.90	0.723
Tail defects (%)	28.20 ± 16.70	28.21 ± 13.86	28.80 ± 13.42	0.988
Total progressive motility (% A+B)	43.64 ± 17.56	44.65 ± 23.05	45.05 ± 19.35	0.974
Fast progressive motility (% A)	2 [1, 41]	2 [1, 5]	2 [0, 3]	0.113
Low progressive motility (% B)	6 [0, 48]	37 [20, 52.50]	45.50 [30, 58.75] ^a^	0.018
Leukocytes (10^6^)	0.491 [0.14, 1.42]	0.70 [0.20, 1.37]	0.80 [0.50, 1.17]	0.152

^1^ Infertile men without human papillomavirus infection. ^2^ Infertile men with single infection high-risk human papillomavirus infection. ^3^ Infertile men with multiple infection high-risk human papillomavirus infection. Normally distributed data are given as mean ± SD; skewed data are given as median ± [25th, 75th] percentiles. The significance tests are the ANOVA test, post hoc Tukey’s for normally distributed variables and the Kruskal–Wallis test for non-distributed variables (*p <* 0.05). ^a^ Infected HPV group vs. non-infected group.

**Table 3:** Oxidative stress biomarkers in semen samples.

Parameter	Non-Infected HPV (*n* = 20)			SI-HR-HPV (*n* = 30)			MI-HR-HPV (*n* = 51)			*p*
	Median	Percentile 25th and 75th	IQR	Median	Percentile 25th and 75th	IQR	Median	Percentile 25th and 75th	IQR	
LPO (nmoles MDA/mg protein)	3.35	2.51, 4.80	2.29	7.43 ^a^	5.55, 8.61	3.22	12.23 ^a,b^	6.89, 12.23	5.48	0.000
8OH-dG (ng/mL)	8.2	1.85, 8.54	6.68	8.34	8.03, 8.80	0.78	8.20	7.70, 8.48	0.79	0.208

Abnormal distributed data are given as median, 25th and 75th percentile and interquartile range (IQR). The significance test is the Kruskal–Wallis test for non-distributed variables (*p <* 0.05). ^a^ Infected HPV group vs. non-infected group; ^b^ SI-HPV group vs. MI-HPV group.

**Table 4:** Spearman correlation analysis between OS biomarkers in seminal plasma with sperm morphology and defects in the infected population (*n* = 81/81, SI-HPV, MI-HPV).

	Correlation	Correlation, *p*-Value
CYP2E1	SI-HR-HPV, MI-HR-HPV	0.841 **, 0.000
CYP2E1	LPO	0.323 **, 0.005
Defects in the tail	8OH-dG	0.246 **, 0.039
CYP2E1	TAC	−0.411 **, 0.000
8OH-dG	% Normal morphology	−0.259 *, 0.030
TAC	Defects in the intermediate piece	−0.283 *, 0.020
Defects in the tail	Defects in the head	−0.512 **, 0.000
Defects in the tail	Defects in the intermediate piece	−0.461 **, 0.000

** Correlation is significant at the 0.01 level (2-tailed). * Correlation is significant at the 0.05 level (2-tailed).

**Table 5:** Cytokines and oxidative stress biomarkers in semen samples.

Cytokine	Non-Infected HPV (*n* = 20)			SI-HPV (*n* = 30)			MI-HPV (*n* = 51)			*p*
	Median	Percentile 25th and 75th	IQR	Median	Percentile 25th and 75th	IQR	Median	Percentile 25th and 75th	IQR	
IFN-γ (pg/mL)	0.000	0.000, 0.000	0.000	398.80 ^a^	206.50, 909	704.38	826.50 ^a^	154.00, 724.00	571.25	0.000
IL-1β (pg/mL)	0.000	0.000, 1.33	1.33	193.98 ^a^	11.33, 1303.00	1289.00	157.16 ^a^	0.000, 328	328	0.000
IL-4 (pg/mL)	6.25	0.000, 26.47	26.47	35.00 ^a^	22.50, 69.00	48.06	41.25 ^a^	17.50, 69.00	52.13	0.000
IL-6 (pg/mL)	0.000	0.000, 12.80	12.80	98.80 ^a^	0.000, 351.20	351.20	203.33 ^a^	36.80, 203.33	166.53	0.001
IL-8 (pg/mL)	395.45	0.000, 395.90	19.3	565.35	272.83, 917.15	644.3	550.00 ^a^	291.63, 1065.64	775.4	0.086

Abnormal distributed data are given as median, 25th and 75th percentile and interquartile range (IQR). The significance test is the Kruskal–Wallis test, *p <* 0.05. ^a^ Infected HPV group vs. non-infected group.

## Data Availability

Not applicable.
